# Psychometric properties of Persian version of depression literacy (D-Lit) questionnaire among general population

**DOI:** 10.1186/s13033-022-00550-x

**Published:** 2022-08-12

**Authors:** Hadi Tehrani, Mahbobeh Nejatian, Mahdi Moshki, Alireza Jafari

**Affiliations:** 1 Social Determinants of Health Research Center, Mashhad University of Medical Sciences, Mashhad, Iran; 2 Department of Health Education and Health Promotion, School of Health, Mashhad University of Medical Sciences, Mashhad, Iran; 3 Social Determinants of Health Research Center, Gonabad University of Medical Sciences, Gonabad, Iran; 4 Department of Health Education and Health Promotion, School of Health, Social Determinants of Health Research Center, Gonabad University of Medical Sciences, Gonabad, Iran; 5 Department of Health Education and Health Promotion, School of Health, Social Development and Health Promotion Research Center, Gonabad University of Medical Sciences, Gonabad, Iran

**Keywords:** Mental Health, Depression literacy (D-Lit), Mental health literacy, Reliability, Validity

## Abstract

**Background:**

The prevalence of depression in society is increasing and there is a need for a suitable tool to assess the health literacy of people in this field. This study was conducted to evaluate the psychometric of the Iranian version of the depression literacy (D-Lit) questionnaire.

**Methods:**

This cross-sectional study was conducted on 845 participants with a proportional stratified sampling method. First, the translation and cultural adaptation of questionnaire was performed. Then, the validity of D-Lit was assessed by face validity, content validity, exploratory factor analysis (EFA), and confirmatory factor analysis (CFA). The reliability of D-Lit was assessed by the Cronbach’s alpha coefficient and McDonald omega coefficient.

**Results:**

Based on the results of EFA, 5 factors emerged with eigenvalues of greater than 1, which accounted for 56.30% of the variance. Based on the results of CFA, one question was deleted and the results of goodness fit indexes confirmed the model. Cronbach’s alpha coefficient and McDonald omega coefficient for D-Lit questionnaire were 0.890 and 0.891, respectively. Finally, D-Lit questionnaire with 21 questions and 5 subscales of Knowledge of the psychological symptoms (5 items), Knowledge about the effectiveness of available treatment methods (4 items), Knowledge about cognitive-behavioral symptoms (6 items), Knowledge about taking medications and their side effects (4 items), and Knowledge of the severity of the disease (2 items) were confirmed.

**Conclusion:**

The results of this psychometric evaluation confirmed the Persian version of D-Lit questionnaire with 21 questions and 5 subscales is an appropriate tool for measuring people's literacy about depression.

**Supplementary Information:**

The online version contains supplementary material available at 10.1186/s13033-022-00550-x.

## Background

World Health Organization (WHO) reported that the number of persons with common mental disorders globally is going up, particularly in lower-income countries. Common mental disorders are depressive disorders and the total number of people living with depression in the world is 322 million [1]. The results of a meta-analysis study on medical students in China showed that the prevalence of depression was 32.74% [2]. The results of a meta-analysis study in Iran showed that the prevalence of depression among Iranians was 49% and the prevalence of very severe, severe, moderate, and mild depression was 5%, 19%, 33%, and 38%, respectively [3]. Depression increases mortality, morbidity, job loss, personal problems, family and country disability, increased suicide, increased risk of heart disease, diabetes, and hypertension [4–8]. Depression literacy (D-Lit) is one of the predictors and indispensable factors for identifying symptoms of depression [9, 10]. The results of various studies have shown that there is a significant relationship between D-Lit and depression and increasing D-Lit can reduce mental illness in individuals [11, 12].

Health literacy definition by Canadian expert panel is “health literacy is the ability to access, understand, evaluate and communicate information as a way to promote, maintain and improve health in a variety of settings across the life-course” [13]. Mental health literacy is the “knowledge and beliefs about mental disorders, which aid their recognition, management, and prevention” [14]. Mental health literacy has several parts of: “(a) knowledge and believes about risk factors and causes; (b) the ability to recognize specific disorders or different types of psychological distress; (c) knowledge and believes about professional help available; (d) knowledge and believes about self-help interventions; (e) knowledge of how to seek mental health information; and (f) attitudes, which facilitates recognition and appropriate help-seeking” [15].

Various studies have shown that there is a relationship between mental health literacy and seeking mental health services and increasing mental health services utilization. People with high D-Lit seek and use more mental health services and also receive specialist help from counselors, psychologists, or mental health clinics [16–19]. The results of the Lam study in 2014 showed that only 16.4% of people with mental health literacy were adequate. The results also showed that the level of mental health literacy is correlated with mental health status, especially youth depression [20]. Baird's study of Older Korean Americans showed that 72% of people had a poor level of D-Lit [21]. In Deen's study, 47% of people had a poor level of D-Lit [22]. According to the results of a study conducted in Iran in 2017, D-Lit was low and only 52.2% and 54% had correct recognition and intention to seek help, respectively [23].

The D-Lit questionnaire is one of the most useful international tools for assessing the status of depressive literacy in communities. This tool was developed by Griffiths et al. In 2004. This tool contains 22 questions that are evaluated in three-point scale of "Yes", "No" and "I do not know". A higher score indicates that the person has a sufficient level of depressive literacy [24]. So far, this questionnaire has been psychometric and used in different cultures and in languages [25–28]. Given the increasing prevalence of mental disorders in society and the importance of early detection of mental illnesses, the importance of an appropriate tool for assessing mental health literacy status, especially depression in the community, seems essential. Due to the lack of appropriate tools for measuring D-Lit in Iranian society, this study was conducted to evaluate the psychometric of the Iranian version of the D-Lit questionnaire in Gonabad city, Iran.

## Methods

The purpose of this cross-sectional study was to determine the psychometric properties of the Iranian version of the D-Lit questionnaire on 845 general populations in Iran, in 2020.

### Sample size

To performing the factor analysis, the sample size of 100 is poor, 200 is fair, 300 is good, 500 is very well, and 1000 and more is considered excellent [29, 30]. In this study, a sample size of 1000 participants was considered for assessment of the structural validity (exploratory factor analysis and confirmatory factor analysis) of the instrument. Due to incomplete information, 155 questionnaires were removed from the analysis, and 845 participants were eventually analyzed.

### Sampling

In this study, samples were selected by the proportional stratified sampling method. In the first stage, the number and population of health centers were determined. In the next step, each health center was considered as a stratum and the sample size was determined according to the population of each stratum (proportional stratified sampling). Samples were then randomly selected from each health center. Health centers in Iran are different from medical centers. Services provided in health centers include preventive services for healthy people. It should be noted that the questionnaire for people who did not read or write was completed by the interviewer. Inclusion criteria were informed consent of the individual to participate in the study, all people 18–65 years old, do not have physical or mental problems, and resident in Gonabad city.

### Instruments

#### Demographic questionnaire

This questionnaire includes questions such as age, sex, level of education, occupation, marital status, and so on.

#### D-Lit Questionnaire

This questionnaire contains 22 questions that assess the level of literacy of people about depression. The questions are rated on a three-point scale of "True", "False" and "I do not know". Each correct answer is assigned a score of 1 and a high score indicates a high level of depressive health literacy [24, 31]. The validity and reliability of this tool were confirmed in Griffiths study and Cronbach's alpha and 3 month test-retest reliability were reported 0.70 and 0.71, respectively [24].

### Translation and cultural adaptation

Translation and cultural adaptation of the English version of the questionnaire into Persian was done based on WHO Guideline [32]. After obtained permission from the original designer of the questionnaire, the English version of the questionnaire was first translated into Persian and adapted by three psychologists. Then a fluent English expert who was not familiar with the specialized English text of psychology translated the questionnaire from Persian to English. The English text of the reverse translation was re-translated into Persian by 3 psychology specialists fluent in English. The final version was prepared and making the necessary corrections. Finally, the translated file was reviewed and approved by six experts in psychology, sociology, and health education and health promotion.

### Validity

Based on the result when the standard questionnaire has been used and translated, quantitative content validity and quantitative face validity were not required for psychometric evaluation of standard questionnaires [33]. In this study, because the standard questionnaire was used, the validity of the questionnaire was assessed by qualitative face validity and qualitative content validity. Also, structural validity was assessed by using exploratory factor analysis (EFA) and confirmatory factor analysis (CFA).

### Qualitative face validity

To get the target group's comments, a face-to-face interview was conducted randomly simple with some people to find the likelihood ambiguity, relevance, appropriateness, and difficulty of each item. Also, questionnaires were provided to 12 health education and psychology experts to assess qualitative face validity.

### Qualitative content validity

At this stage to conducted content validity, a questionnaire was provided to 12 experts in health education and psychology to investigate grammar, the importance of items, the use of appropriate words, placement of items in the proper place, and the time required to complete the tool. It was reviewed and consulted with members of the research team to make the necessary changes to the instrument.

### EFA

EFA was performed using SPSS _V_._22_ software. In this study eigenvalues above 1, minimum factor loading of 0.4, maximum 25 repetitions of rotation and a scree map were used to determine the number of potential underlying factors [34, 35]. Also, two tests of the Kaiser-Meyer-Olkin (KMO) and Bartlett’s Test of Sphericity were used to investigate the appropriate sample size in EFA [36, 37].

### CFA

The CFA was performed to examine whether the data fit the theoretical model. Before the CFA, the outlier’s data were eliminated by using the Mahalanobis statistical index. Also, the normality of data was evaluated using skewness and kurtosis. CFA was performed using AMOS _V.24_ software. The goodness-of-fit of the model was assessed using chi-square indicators (χ^2^), chi-square ratio to degree of freedom (χ^2^/df), root mean square error of approximation (RMSEA), root mean square residual (RMR), parsimonious normed fit index (PNFI), parsimony comparative fit index (PCFI), adjusted goodness of fit index (AGFI), goodness of fit index (GFI), parsimony goodness of fit index (PGFI), incremental fit index (IFI), parsimonious normed fit index (PNFI), and comparative fit index (CFI) [38–40]. The model was considered to be a good fit if the (χ^2^/df) < 5, RMSEA and RMR < 0.08, AGFI > 0.8, PCFI, PNFI, and PGFI > 0.5, and other indices (GFI, GFI, IFI) more than 0.9 [38–41].

### Reliability

To assess the internal consistency of the D-Lit questionnaire and each of the subscales separately, the Cronbach's alpha coefficient was used. Based on the results, the Cronbach’s alpha coefficient of ranging from 0.70 to 0.95 was the acceptance criterion for the internal reliability of the instrument [42, 43]. The software of JASP _V.0.11.1_ was used to calculate the McDonald’s omega coefficient.

## Results

### Descriptive characteristics

According to the results of this study, the mean (± standard deviation) age of participants was 30.86 (± 10.11). Of the participants, 57.9% (n = 485), 42.1% (n = 353), 68.8% (n = 574), and 31.1% (n = 259) were female, male, married, and single, respectively. In this study, 2.3%, 30.8%, 56.2% and 9.8% of the participants had elementary education, diploma, associate or bachelor's degree, and master's degree/high degree, respectively. Most people's jobs were self-employed (46.8%) and employed (31.6%). Other demographic information can be seen in Table 1.

**Table 1:** Frequency distribution of demographic information (n = 845)

Variables		N	%
Sex	Male	353	42.1
	Female	485	57.9
Marital status	Marriage	574	68.9
	Single	259	31.1
Education level	Elementary	26	3.2
	Diploma	251	30.8
	Associate or Bachelor's degree	458	56.2
	Master's degree or High degree	80	9.8
Residence	Urban	615	78.8
	Rural	165	21.2
Job	Housewife	129	16
	Employed	254	31.6
	Self-employed	276	46.8
	Unemployed	45	5.6

### Translation and cultural adaptation

During the process of translation and cultural adaptation, the topics mentioned in the original questionnaire were not excluded due to the matching of the topics with the culture of the Iranian people.

### Validity

In this section, qualitative face validity and qualitative content validity were assessed. In qualitative face validity, 4 items were modified. In qualitative content validity, 6 items were modified.

### EFA

**Fig. 1:**
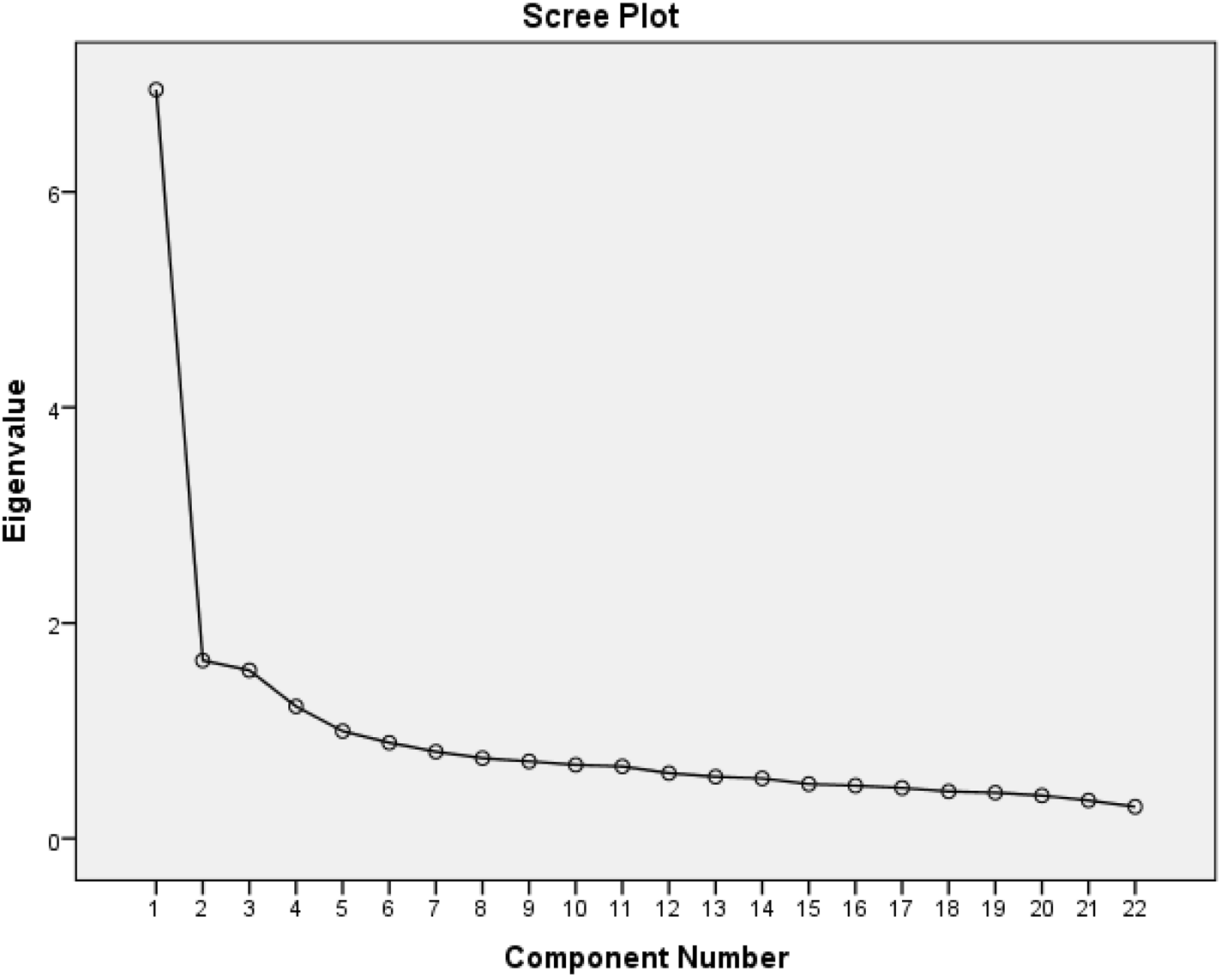
Scree plot of the factor analysis of the Persian version of D-Lit questionnaire

At this stage, before conducting EFA, sampling adequacy was first evaluated using KMO and Bartlett's Test of Sphericity, and sampling adequacy were confirmed (KMO = 0.911 and Bartlett’s test: χ^2^ = 6103.662, df = 231, p < 0.001). Based on the results of EFA five factors emerged with eigenvalues of greater than 1, which accounted for 56.30% of the variance (Table 2 and Table 3). Also, these results were illustrated in Fig. 1 as a scree plot.

**Table 2:** The five-factor structure of the D-Lit questionnaire

Total variance explained									
Component	Initial eigenvalues			Extraction sums of squared loadings			Rotation sums of squared loadings		
	Total	% of Variance	Cumulative %	Total	% of Variance	Cumulative %	Total	% of Variance	Cumulative %
1	6.950	31.592	31.592	6.950	31.592	31.592	3.150	14.317	14.317
2	1.652	7.511	39.103	1.652	7.511	39.103	2.899	13.177	27.494
3	1.561	7.097	46.200	1.561	7.097	46.200	2.619	11.906	39.401
4	1.226	5.575	51.775	1.226	5.575	51.775	2.142	9.736	49.137
5	0.997	4.530	56.305	0.997	4.530	56.305	1.577	7.168	56.305
6	0.889	4.040	60.345						
7	0.806	3.662	64.007						
8	0.745	3.387	67.394						
9	0.715	3.252	70.646						
10	0.683	3.104	73.750						
11	0.669	3.041	76.791						
12	0.606	2.756	79.547						
13	0.573	2.603	82.150						
14	0.558	2.535	84.685						
15	0.504	2.293	86.978						
16	0.490	2.229	89.207						
17	0.469	2.132	91.339						
18	0.436	1.982	93.322						
19	0.425	1.930	95.252						
20	0.398	1.808	97.060						
21	0.353	1.604	98.664						
22	0.294	1.336	100.000						

Extraction Method: Principal Component Analysis

**Table 3:** Rotated Factor Matrix of the D-Lit questionnaire

Rotated component matrix^a^					
Items	Component				
	1	2	3	4	5
Q7	0.784				
Q4	0.744				
Q8	0.724				
Q2	0.634				
Q11	0.631				
Q17		0.801			
Q16		0.765			
Q18		0.754			
Q12		0.416			
Q13		0.367			
Q5			0.648		
Q3			0.632		
Q6			0.620		
Q1			0.614		
Q9			0.454		
Q10			0.350		
Q22				0.768	
Q21				0.706	
Q20				0.701	
Q19				0.540	
Q14					0.765
Q15					0.538

Extraction Method: Principal Component Analysis. Rotation Method: Varimax with Kaiser Normalization

^a^Rotation converged in 7 iterations

### CFA

**Fig. 2:**
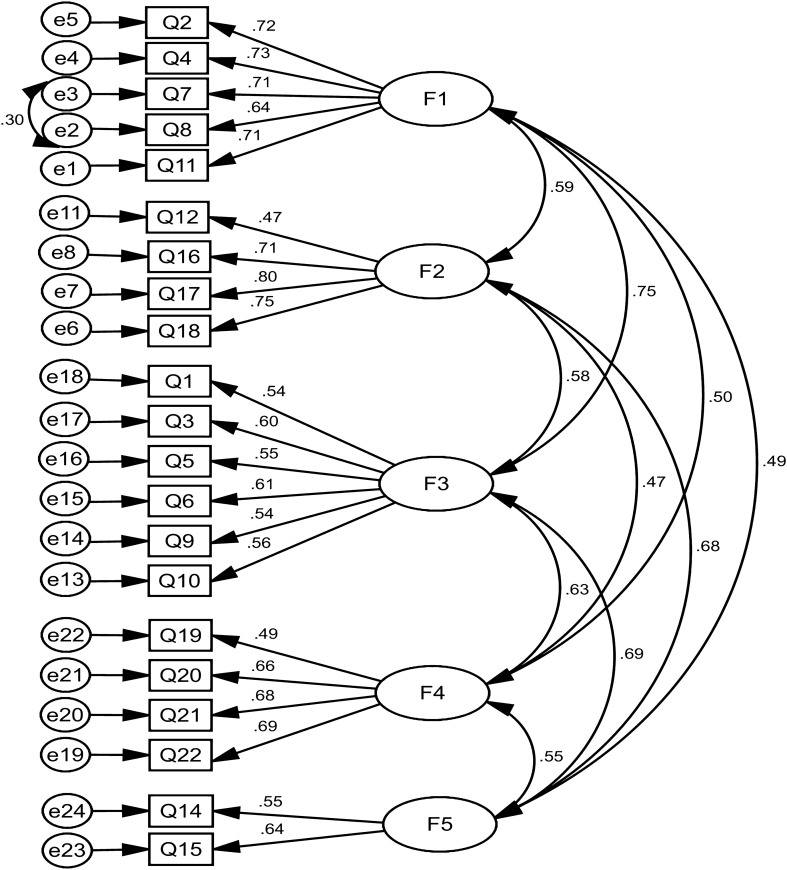
Standardized parameter estimates for the factor structure of the D-Lit questionnaire (All factor loadings are significant at p < 0.001, F1: Knowledge of the psychological symptoms, F2: Knowledge about the effectiveness of available treatment methods, F3: Knowledge about cognitive-behavioral symptoms, F4: Knowledge about taking medications and their side effects, F5: Knowledge of the severity of the disease)

At this stage, five factors obtained in EFA were investigated using CFA. Based on the results of CFA, the indices confirmed the model (Table 4). In this stage, one question was deleted. The results of some goodness fit indexes for this proposed model were: χ^2^/df = 3.635, RMSEA = 0.056, PNFI = 0.754, GFI = 0.932, and CFI = 0.917 (Table 4). Also, the factor loading of all items were mentioned in Table 5 and Fig. 2.

**Table 4:** The model fit indicators of the D-Lit questionnaire

Goodness of fit indices	CFA	Acceptable value
χ^2^	646.984	–
df	178	–
χ^2^/df	3.635	< 5
p-value	< 0.001	> 0.05
CFI	0.917	> 0.9
IFI	0.917	> 0.9
GFI	0.932	> 0.9
AGFI	0.911	> 0.8
RMSEA	0.056	< 0.08
RMR	0.031	< 0.08
PNFI	0.754	> 0.5
PCFI	0.777	> 0.5
PGFI	0.718	> 0.5

**Table 5:** Factor loadings of the D-Lit questionnaire in the CFA among general population

Subscale	Items	Factor loadings (Standardized regression weights)
F1: Knowledge of the psychological symptoms	Q2: People with depression may feel guilty when they have done nothing wrong. (True)	0.716
	Q4: Loss of confidence and low self-esteem may be a sign of depression. (True)	0.729
	Q7: Too little or too much sleep can be a symptom of depression. (True)	0.709
	Q8: Eating too much or losing interest in food may be a symptom of depression. (True)	0.640
	Q11: People may move more slowly or become agitated due to their depression. (True)	0.713
F2: Knowledge about the effectiveness of available treatment methods	Q12: Clinical psychologists can prescribe antidepressant medications. (False)	0.470
	Q16: Many treatments for depression are more effective than antidepressant medications. (False)	0.711
	Q17: The effects of counseling are similar to those of cognitive- behavioral therapies for depression. (False)	0.797
	Q18: The effect of cognitive-behavioral therapies is the same as that of antidepressant medications for mild to moderate depression. (True)	0.754
	*Q13: Having a moderate depression can disrupt one's life as much as multiple sclerosis or deafness*	Deleted*
F3: Knowledge about cognitive-behavioral symptoms	Q1: People with depression often speak sporadically and irrelevantly. (False)	0.541
	Q3: Reckless and foolhardy behavior is a common symptom of depression. (False)	0.602
	Q5: Not walking on cracked and broken sidewalks may be a symptom of depression. (False)	0.549
	Q6: People with depression often hear sounds that are not normally heard. (False)	0.610
	Q9: Depression does not affect your memory and concentration. (False)	0.536
	Q10: Having several distinct personalities can be a symptom of depression. (False)	0.562
F4: Knowledge about taking medications and their side effects	Q19: Of all the alternative and lifestyle therapies for depression, taking vitamins are the most beneficial. (False)	0.495
	Q20: People with depression should stop taking antidepressant medications as soon as they feel better. (False)	0.658
	Q21: Antidepressant medications are addictive. (False)	0.682
	Q22: Antidepressant medications are usually rapid-acting. (False)	0.693
F5: Knowledge of the severity of the disease	Q14: Most people with depression need to be hospitalized. (False)	0.553
	Q15: Many celebrities have suffered from depression. (True)	0.639

^*^This question was deleted in confirmatory factor analysis stage

### Reliability

Cronbach's alpha coefficient for D-Lit questionnaire and subscales of Knowledge of the psychological symptoms (F1), Knowledge about the effectiveness of available treatment methods (F2), Knowledge about cognitive-behavioral symptoms (F3), Knowledge about taking medications and their side effects (F4), and Knowledge of the severity of the disease (F5) were 0.890, 0.837, 0.767, 0.739, 0.723, and 0.522, respectively. McDonald omega coefficient for D-Lit questionnaire and subscales of F1, F2, F3, F4, and F5 were 0.891, 0.838, 0.779, 0.740, 0.728, and 0.522 respectively (Table 6). The Persian version of the D-Lit questionnaire is in the supplementary files (Additional file 1).

**Table 6:** Descriptive statistics of the D-Lit questionnaire subscale scores among general population

Subscales	Item	Range	Cronbach’s alpha coefficient	McDonald omega coefficient
F1: Knowledge of the psychological symptoms	5	5–15	0.837	0.838
F2: Knowledge about the effectiveness of available treatment methods	4	4–12	0.767	0.779
F3: Knowledge about cognitive-behavioral symptoms	6	6–18	0.739	0.740
F4: Knowledge about taking medications and their side effects	4	4–12	0.723	0.728
F5: Knowledge of the severity of the disease	2	2–6	0.522	0.522
All subscales of D-Lit questionnaire	21	21–63	0.890	0.891

## Discussion

This study aimed to psychometrically assess the D-Lit questionnaire. Based on the results of EFA, the questionnaire had 5 subscales with specific values greater than 1, which was able to predict 56.30% variance. In the CFA stage, these 5 factors were examined and one question was removed and finally, the questionnaire with 5 factors and 21 questions was approved. The reliability of the questionnaire was assessed using Cronbach's alpha and Omega-McDonald's coefficient, which were 0.890 and 0.891, respectively, for all questions.

In Griffiths et al. study, the validity and reliability of the questionnaire were examined and Cronbach's alpha and 3 month test-retest reliability were reported 0.70 and 0.71, respectively [24]. The Griffiths study showed that this questionnaire is a suitable tool to assess the depression literacy status. Also, results showed that depressive literacy may be useful in reducing social stigma in people with depression [24]. In a study conducted by Wang with the aim of psychometric evaluation of the D-Lit questionnaire on Chinese people, the results showed that Cronbach's alpha and content validity were 0.885 and 0.989, respectively, and the Chinese version of this questionnaire had acceptable validity and reliability for assessing the knowledge of people about depression [44]. In a study conducted by Arafat to psychometrically evaluate the Bangla version of the D-Lit questionnaire, the Cronbach's alpha was 0.77. After validity and reliability, according to the expert’s opinion, 3 questions were removed due to cultural equivalence and one question was added to the questionnaire and finally, the 20-question version of the questionnaire with one factor was confirmed [27]. In a study aimed at psychometric evaluation of the Arabic version of the D-Lit questionnaire, Cronbach's alpha coefficient, split-half test, test-retest, and Spearman's correlation were 0.78, 0.71, 0.92, and 0.91, respectively. None of the questionnaire questions were removed and the 22-item version of the questionnaire was approved by one factor [25].

In the Ibrahim study conducted in the Malaysian population, 5 questions were removed from the D-Lit questionnaire and Cronbach's alpha of the questions was 0.6 [45]. The results of the Oliffe study in the Canadian male population showed that Cronbach's alpha of D-Lit questionnaire was 0.74 [46]. A study of the Korean American population by Bernstein showed that the Cronbach's alpha of the D-Lit questionnaire was 0.81 [47]. In the Ram study on healthcare profession students, the results showed that Cronbach's alpha of the questionnaire was 0.74 [48]. The results of the Kiropoulos study in Australia showed that Cronbach's alpha levels of D-Lit questionnaire in the Greek and Italian populations living in Australia were 0.88 and 0.99, respectively [49]. In a study conducted by Bonabi in the United States, the Cronbach's alpha of the D-Lit questionnaire was 0.89 [50].

A study conducted by Jeong on Korean pregnant women showed that the content validity of the parental D-Lit scale was 0.875. During the validity and reliability, 4 questions were removed from the questionnaire. Results of EFA showed that the questionnaire has 3 factors of misperceptions about depression and its treatment, knowledge about the treatment of depression, and knowledge about depression. The results of CFA showed that the RMSEA, CFI and χ^2^/df indices were 0.056, 0.813, and 1.44, respectively, which confirmed these three factors. Finally, the questionnaire was approved with 18 questions and three factors [26].

In this study, the first factor was “*knowledge of the psychological symptoms”*, which was confirmed by 5 questions, standard regression coefficient 0.640 to 0.729, Cronbach's alpha coefficient 0.837, and omega McDonald coefficient 0.838. knowledge about the psychological symptoms of depression is effective in seeking mental health services [51]. The results of a study showed that people with higher levels of depression had less knowledge about recognizing depression and seeking professional help-seeking than people with low levels of depression [16]. The results of a study showed that most people had little knowledge about depression and had poor diagnostic ability about depression [52].

The second factor in this study was the “*knowledge about the effectiveness of available treatment methods*”, which were confirmed by 4 questions, the standard regression coefficient 0.470 to 0.797, Cronbach's alpha coefficient of 0.767, and the omega McDonald coefficient of 0.779. Most people with mental disorders do not use mental health services because they are unaware of the available treatments [53]. Also, the most important reason for patients with mental disorders to delay receiving treatment is the lack of knowledge about available effective treatments. Therefore, having sufficient knowledge about effective treatment methods for mental disorders seems to be necessary [54]. Treatment of depression in the early stages reduces the symptoms of the disease and prevents of severe depression [55].

In the present study, the third factor was “*knowledge about cognitive-behavioral symptoms”* which was confirmed by 6 questions, standard regression coefficient 0.536 to 0.610, Cronbach's alpha coefficient 0.739, and omega McDonald coefficient 0.740. Another aspect of depression is cognitive-behavioral problems and major cognitive symptoms of depression include difficulty concentrating and decision problems [56]. Having these symptoms can cause the person to not be able to diagnose the problem and not pay attention to the available information. Therefore, knowledge about the cognitive-behavioral symptoms of depression is essential for the early diagnosis of diseases [57, 58].

The fourth factor was *“knowledge about taking medications and their side effects”*, which was confirmed by 4 questions, standard regression coefficient 0.495–0.693, Cronbach's alpha coefficient 0.723, and Omega McDonald coefficient 0.728. Opinions differ on the effectiveness of antidepressants in relieving depressive symptoms. Antidepressants, like many other treatments, may help in some cases and not be useful in others. These medications of depression can also have side effects similar to other medications. People with depression should be sufficiently knowledge of this and receive the necessary information from their physician about the pros and cons of antidepressants [59].

In this study, the fifth factor was “*knowledge of the severity of the disease”*, which was confirmed by 2 questions, standard regression coefficient of 0.533 and 0.639, Cronbach's alpha coefficient of 0.522, and Omega-McDonald coefficient of 0.522. Another important factor is having the right knowledge about the disease. A study finding showed that many public people are not able to recognize specific disorders or different types of mental disorders [15]. The results of a study in China showed that people who more learned about mental disease had more knowledge about the mental health [60]. A randomized controlled trial showed that the intervention of web-based D-Lit had a significant decrease in the stigmatizing attitudes of people who experienced depression with severe symptoms [61]. Therefore, having accurate and reliable information about the disease is essential and allows the person to refer to the systems providing mental health services without fear of treatments and use the available treatments [62–64]. One of the strengths of this study was that the study was conducted with a high sample size. Also, this study was conducted on the general population and this questionnaire can be used for different target groups. One of the limitations of this study was that the reliability of the study was not performed using the test-retest method. Due to the COVID-19 pandemic, it was not possible to perform the test-retest. Another limitation of this study was that the information was completed using a questionnaire and self-report and may have some errors. In this study, people with a clear diagnosis of mental health problems were not included in the study because people with mental health disorders due to referral and follow-up treatment may have high levels of depression literacy.

## Conclusions

Based on the results of EFA and CFA, the Persian version of the D-Lit questionnaire with 21 questions and 5 subscales of Knowledge of the psychological symptoms (5 items), Knowledge about the effectiveness of available treatment methods (4 items), Knowledge about cognitive-behavioral symptoms (6 items), Knowledge about taking medications and their side effects (4 items), and Knowledge of the severity of the disease (2 items) were confirmed. Finally, this questionnaire is an appropriate and convenient tool for measuring people's knowledge about depression. Knowing the state of mind of people about depression can help design prevention programs for different age groups.

## Supplementary Information

**Additional file 1.** Persian version of Depression Literacy (D-Lit) questionnaire (21 Items)

## Data Availability

All data generated or analysed during this study are included in this published article.

## Abbreviations

D-Lit: Depression literacy
EFA: Exploratory factor analysis
CFA: Confirmatory factor analysis
KMO: Kaiser-Meyer-Olkin
χ^2^: Chi-square indicators
df: Degree of freedom
RMSEA: Root mean square error of approximation
RMR: Root mean square residual
PNFI: Parsimonious normed fit index
PCFI: Parsimony comparative fit index
AGFI: Adjusted goodness of fit index
GFI: Goodness of fit index
PGFI: Parsimony goodness of fit index
IFI: Incremental fit index
CFI: Comparative fit index
WHO: World Health Organization
F1: Knowledge of the psychological symptoms
F2: Knowledge about the effectiveness of available treatment methods
F3: Knowledge about cognitive-behavioral symptoms
F4: Knowledge about taking medications and their side effects
F5: Knowledge of the severity of the disease
