# Supplementary material for: Psychometric properties of Persian version of depression literacy (D-Lit) questionnaire among general population
Source: Int J Ment Health Syst. 2022 Aug 12;16:40. doi: 10.1186/s13033-022-00550-x (PMC9372931; doi:10.1186/s13033-022-00550-x)
Supplement: Supplementary file 1 — Additional file 1. Persian version of Depression Literacy (D-Lit) questionnaire (21 Items) [file 13033_2022_550_MOESM1_ESM.doc]

**Additional file 1**

- **Persian version of Depression Literacy (D-Lit) questionnaire (21 Items)**

| **ردیف** | **سوالات** | **فاکتور ها** |
| --- | --- | --- |
| 1 | افراد مبتلا به افسردگی ممکن است زمان که هیچ کار اشتباهی نکرده اند، احساس گناه کنند. (درست) | **فاکتور اول:**  **(آگاهی از علایم روانی افسردگی)** |
| 2 | از دست دادن اعتماد به نفس و عزت نفس پایین ممکن است نشانه افسردگی باشد. (درست) |
| 3 | از جمله نشانه های افسردگی می تواند خواب بیش از حد یا خیلی کم باشد. (درست) |
| 4 | خوردن بیش از حد یا از دست دادن علاقه به مواد غذایی ممکن است نشانه افسردگی باشد. (درست) |
| 5 | از جمله نتایج افسردگی ممکن است خیلی آهسته حرکت کردن و مضطرب شدن افراد باشد. (درست) |
| 6 | روانشناسان بالینی می توانند داروهای ضد افسردگی را تجویز کنند. (نادرست) | **فاکتور دوم:**  **(آگاهی از اثربخشی روش های درمانی موجود)** |
| 7 | بسیاری از روش های درمانی افسردگی تاثیر بیشتری نسبت به دارو های ضد افسردگی دارند. (نادرست) |
| 8 | تاثیر مشاوره درمانی همانند تاثیر درمان های شناختی- رفتاری برای افسردگی است. (نادرست) |
| 9 | تاثیر درمان های شناختی- رفتاری همانند تاثیر داروهای ضد افسردگی برای افسردگی خفیف تا متوسط است. (درست) |
| 10 | افراد مبتلا به افسردگی اغلب بصورت پراکنده و بی ربط صحبت می کنند. (نادرست) | **فاکتور سوم:**  **(آگاهی از علایم شناختی-رفتاری افسردگی)** |
| 11 | رفتار جسورانه و عاری از مسئولیت یک علامت معمول افسردگی است. (نادرست) |
| 12 | قدم نگذاشتن روی قسمت های ترک خوره و شکسته پیاده رو ممکن است نشانه افسردگی باشد. (نادرست) |
| 13 | افرادی که مبتلا به افسردگی هستند اغلب صداهایی را می شنوند که در حالت طبیعی شنیده نمی شوند. (نادرست) |
| 14 | افسردگی حافظه و تمرکز شما را تحت تاثیر قرار نمی دهد. (نادرست) |
| 15 | داشتن چندین شخصیت متمایز ممکن است نشانه ای از افسردگی باشد. (نادرست) |
| 16 | از بین همه درمان های طب مکمل/جایگزین و شیوه زندگی برای افسردگی، مصرف ویتامین ها بیشتر مفید خواهد بود. (نادرست) | **فاکتور چهارم:**  **(آگاهی نسبت به مصرف داروها و عوارض آن)** |
| 17 | افراد مبتلا به افسردگی باید مصرف داروهای ضد افسردگی را به محض احساس بهتر شدن قطع کنند. (نادرست) |
| 18 | داروهای ضد افسردگی اعتیاد آور هستند. (نادرست) |
| 19 | داروهای ضد افسردگی معمولا بعد از مصرف بلافاصله تاثیر خود را نشان می دهند. (نادرست) |
| 20 | اکثر افراد مبتلا به افسردگی باید در بیمارستان بستری شوند. (نادرست) | **فاکتور پنجم:**  **(آگاهی از شدت بیماری)** |
| 21 | بسیاری از افراد معروف از افسردگی رنج برده اند. (درست). |
